# Supplementary material for: Variation in Dysphagia Assessment and Management in Acute Stroke: An Interview Study
Source: Geriatrics (Basel). 2019 Oct 25;4(4):60. doi: 10.3390/geriatrics4040060 (PMC6960646; doi:10.3390/geriatrics4040060)
Supplement: Supplementary file 1 [file geriatrics-04-00060-s001.pdf]

Variation in dysphagia assessment and management in acute stroke: An interview study

Sabrina A. Eltringham [1,2,*], Craig J. Smith [3], Sue Pownall [1], Karen Sage [2], Ben Bray [4]

[1] Speech and Language Therapy Department, Sheffield Teaching Hospitals NHS Foundation Trust, Sheffield, S10 2JF, UK
[2] Faculty of Health and Wellbeing, Sheffield Hallam University, Sheffield, S10 2BP, UK
[3] Division of Cardiovascular Sciences, University of Manchester, Manchester Centre for Clinical Neurosciences, Salford Royal NHS Foundation Trust, Manchester Academic Health Science Centre, Manchester, M6 8HD, UK
[4] School of Population Health and Environmental Sciences, King's College London, London, SE1 1UL, UK
* Correspondence: s.a.eltringham@shu.ac.uk

SUPPLEMENTARY MATERIAL

Table 1 – Summary of Royal College of Physicians Clinical Guideline for Stroke (2016) specifically related to dysphagia screening, assessment and oral care.

Chapter	Recommendation
**Acute Care**<br>**Recommendation 3.10.1 E**	Patients with acute stroke should have their swallowing screened, using a validated screening<br>tool, by a trained healthcare professional within four hours of arrival at hospital and before<br>being given any oral food, fluid or medication.
**Recommendation 3.10.1 F**	Until a safe swallowing method is established, patients with dysphagia after acute stroke<br>should:<br>– Be immediately considered for alternative fluids;<br>– Have a comprehensive specialist assessment of their swallowing;<br>– Be considered for nasogastric tube feeding within 24 hours;<br>– Be referred to a dietitian for specialist nutritional assessment, advice and monitoring;<br>– Receive adequate hydration, nutrition and medication by alternative means.
**Recovery and Rehabilitation**<br>**Recommendation 4.11.1 A**	People with stroke, especially those who have difficulty swallowing or are tube fed, should have mouth care at least 3 times a day including:<br>– Brushing of teeth and cleaning of gums with a suitable cleaning agent (toothpaste and/or<br>chlorhexidine dental gel), for which an electric toothbrush should be considered;<br>– Removal of excess secretions;

– Application of lip balm.

Table 2: Topic guide for staff interviews

Thank you for agreeing to take part in this interview. I am interested to hear about what happens in the first few days when patients are admitted after a stroke? It might help if you remember the last person you cared who was admitted on the stroke pathway…..

Questions for All Staff Groups

What is the admission process and pathway for acute stroke patients (e.g., admission to the emergency department, transfer to the stroke bed/hyper acute stroke unit (HASU), direct admission to stroke bed)?

What happens to patients before they get a swallow screen?

What happens to patients admitted overnight or at weekends, or for patients who are outside HASU?

What do you think are the main factors, which contribute to delays in a) screening and b) assessment?

Is there an integrated team approach for the management of patients with dysphagia?

Does a speech and language therapist (SLT) or dysphagia trained practitioner attend daily ward round with the multidisciplinary team (MDT)?

Do you have access to a dietician?

What do you do if the patient is nil by mouth (NBM) and requires alternative feeding overnight or at the weekend?

What happens if there are accidents or errors (e.g., fed despite NBM)?

Specific Questions for Doctors and Ward Sisters

What is the practice in terms of nasogastric (NG) tube insertion?

Who inserts the nasogastric tube (NGT)?

How many NGT insertions are permitted?

How many staff are trained to insert an NGT?

Do you use NGT bridles?

What feeding protocol do you use during <72 hours?

Do you have an oral care policy?

What does this consists of?
How frequently is this carried out?
Are dysphagia patients managed differently?
Do you have access to professional oral care?
Do you use selective decontamination of the digestive tract?
What is your approach to mobilization during <72 hours?
What is your approach to positioning during NG feeding and at mealtimes? Have there been any changes since the head post-trial?
Do you use any medications to reduce risk of stroke-associated pneumonia (SAP) (acid suppressive medications, antiemetic, angiotensin-converting enzyme inhibitors, antibiotics)?
Are there any confounding factors, which may impact on use of these medications?

Specific questions for SLT Stroke Team Leaders, Speech and Language Therapists and Trained Dysphagia Screeners

How do you identify which patients need a) dysphagia screen and b) SLT swallowing assessment?
How do you prioritize which patients are a) screened and b) assessed first?
How long does it usually take to a) screen and b) assess a patient?
What dysphagia screening protocol do you use?
Who typically undertakes the dysphagia screen?
After the dysphagia screen who manages the patient's swallow?
Do you use a validated bedside swallow assessment such as Mann Assessment of Swallowing Ability?
How frequently is a patient's swallow reviewed?
What level of supervision is there for dysphagia patients?
What types of dysphagia management strategies are used?
When do you initiate swallowing therapy?
Do you have access to videofluroscopy and/or fibreoptic endoscopic evaluation of swallowing (FEES)?
How frequently is this used during first 7 days of admission?
How are the findings and recommendations of a) the screen and b) assessment communicated with other members of the MDT?
How are the findings and recommendations communicated with patients and family members?
What does the SLT swallow assessment involve?

Closing question—Is there anything in those first 72 hours, which you think could be handled differently? Or anything you would like to tell me that I haven't asked?

Table 3 – Participant characteristics

Participant ID	Professional Role	Years Professionally Qualified	IDF Competency
**H1P1**	Stroke Specialist Nurse	27 yrs.	Specialist Level
**H1P2**	Charge Nurse	10 yrs.	Foundation Level
**H1P3**	Doctor	18 yrs.	N/A
**H2P1**	SLT	17 yrs.	Specialist Level
**H2P2**	Doctor	14 yrs.	N/A
**H2P3**	Stroke Specialist Nurse	14 yrs.	Foundation Level
**H3P1**	Doctor	23 yrs.	N/A
**H3P2**	SLT	10 yrs.	Specialist Level
**H3P4**	Rapid Access Protocol Nurse	4.5 yrs.	Foundation Level
**H4P1**	Doctor	20 yrs.	N/A
**H4P3**	Clinical Lead for Stroke Nurse Practitioners	16 yrs.	Foundation Level
**H4P4**	Stroke SLT Clinical Lead	16 yrs.	Specialist Level
**H5P1**	Stroke SLT Clinical Lead	8 yrs.	Specialist Level
**H5P2**	Practice Educator	11 yrs.	Foundation Level
**H5P4**	SLT	5 yrs.	Specialist Level

SCN—strategic clinical network, IDF—inter professional dysphagia framework.

Table 4 – Type of dysphagia screening protocol

Hospital ID	Type of DSP	Screen components	Consistencies
**H1**	Locally developed tool	Pre-screen check fluids and diet	>100 ml Level 0 H20, Level 4 puree, Level 6 soft and bite sized, Level 7 regular ETC, Level 7 regular
**H2**	Locally developed tool	Pre-screen check fluids only	>3 sips Level 0 H20, >3 sips Level 3 moderately thick fluids
**H3**	Locally developed tool	Pre-screen check basic screen advanced Screen	Level 0 H20, Level 7 ETC Level 3 moderately thick fluids, Level 4 puree
**H4**	Locally developed tool	Part A—Pre-screen tasks, Part B—H20, Part C—diet	>50mls Level 0 H20, Level 7 regular ETC or Level 7 regular
**H5**	Locally developed tool	Pre-screen check fluids only	>cup Level 0 H20

At the time of the interviews some hospitals were in the process of transitioning to the International Dysphagia Diet Standardisation Initiative (IDDSI) descriptors.[2] In two hospitals they were using a combination of the IDDSI descriptors for fluid consistencies and the National Descriptors for diet consistencies. For comparative purposes in Table 1 National descriptors have been converted to the IDDSI descriptors.

In Hospital 3 patients who were screened on Level 7 easy to chew (ETC) diet would be automatically upgraded to Level 7 regular diet after 24 hrs. In Hospital 5 if patients passed the screening they would be served a Level 7 regular diet at the next mealtime.

References

1. Boaden, E. & Davies, S. Inter Professional Dysphagia Framework https://docs.google.com/a/nihr.ac.uk/viewer?a=v&pid=sites&srcid=bmloci5hYy51a3xkeXNwaGFnaWEtdG9vbGtpdHxneDo2ZDFhN2IzYjQ4MDA5ZTQy Accessed May 2019.
2. The International Dysphagia Diet Standardisation Initiative 2016 https://iddsi.org/framework/.

Standards for Reporting Qualitative Research (SRQR)*

http://www.equator-network.org/reporting-guidelines/srqr/

Title and abstract

	Page/line no(s).
**Title** - Concise description of the nature and topic of the study Identifying the study as qualitative or indicating the approach (e.g., ethnography, grounded theory) or data collection methods (e.g., interview, focus group) is recommended	1
**Abstract** - Summary of key elements of the study using the abstract format of the intended publication; typically includes background, purpose, methods, results, and conclusions	2

Introduction

**Problem formulation** - Description and significance of the problem/phenomenon studied; review of relevant theory and empirical work; problem statement	2-4
**Purpose or research question** - Purpose of the study and specific objectives or questions	4

Methods

**Qualitative approach and research paradigm** - Qualitative approach (e.g., ethnography, grounded theory, case study, phenomenology, narrative research) and guiding theory if appropriate; identifying the research paradigm (e.g., postpositivist, constructivist/ interpretivist) is also recommended; rationale**	4
**Researcher characteristics and reflexivity** - Researchers' characteristics that may influence the research, including personal attributes, qualifications/experience, relationship with participants, assumptions, and/or presuppositions; potential or actual interaction between researchers' characteristics and the research questions, approach, methods, results, and/or transferability	5, 17-18
**Context** - Setting/site and salient contextual factors; rationale**	4
**Sampling strategy** - How and why research participants, documents, or events were selected; criteria for deciding when no further sampling was necessary (e.g., sampling saturation); rationale**	4
**Ethical issues pertaining to human subjects** - Documentation of approval by an appropriate ethics review board and participant consent, or explanation for lack thereof; other confidentiality and data security issues	5
**Data collection methods** - Types of data collected; details of data collection procedures including (as appropriate) start and stop dates of data collection and analysis, iterative process, triangulation of sources/methods, and modification of procedures in response to evolving study findings; rationale**	5

**Data collection instruments and technologies** - Description of instruments (e.g., interview guides, questionnaires) and devices (e.g., audio recorders) used for data collection; if/how the instrument(s) changed over the course of the study	5
**Units of study** - Number and relevant characteristics of participants, documents, or events included in the study; level of participation (could be reported in results)	4, Supplementary material
**Data processing** - Methods for processing data prior to and during analysis, including transcription, data entry, data management and security, verification of data integrity, data coding, and anonymization/de-identification of excerpts	5
**Data analysis** - Process by which inferences, themes, etc., were identified and developed, including the researchers involved in data analysis; usually references a specific paradigm or approach; rationale**	5
**Techniques to enhance trustworthiness** - Techniques to enhance trustworthiness and credibility of data analysis (e.g., member checking, audit trail, triangulation); rationale**	5

Results/findings

**Synthesis and interpretation** - Main findings (e.g., interpretations, inferences, and themes); might include development of a theory or model, or integration with prior research or theory	6-15
**Links to empirical data** - Evidence (e.g., quotes, field notes, text excerpts, photographs) to substantiate analytic findings	6-15

Discussion

**Integration with prior work, implications, transferability, and contribution(s) to the field -** Short summary of main findings; explanation of how findings and conclusions connect to, support, elaborate on, or challenge conclusions of earlier scholarship; discussion of scope of application/generalizability; identification of unique contribution(s) to scholarship in a discipline or field	16-18
**Limitations** - Trustworthiness and limitations of findings	17-18

Other

**Conflicts of interest** - Potential sources of influence or perceived influence on study conduct and conclusions; how these were managed	N/A
**Funding** - Sources of funding and other support; role of funders in data collection, interpretation, and reporting	Entered separately in the submission process

*The authors created the SRQR by searching the literature to identify guidelines, reporting standards, and critical appraisal criteria for qualitative research; reviewing the reference lists of retrieved sources; and contacting experts to gain feedback. The SRQR aims to improve the transparency of all aspects of qualitative research by providing clear standards for reporting qualitative research.

**The rationale should briefly discuss the justification for choosing that theory, approach, method, or technique rather than other options available, the assumptions and limitations implicit in those choices, and how those choices influence study conclusions and transferability. As appropriate, the rationale for several items might be discussed together.

Reference:

O'Brien BC, Harris IB, Beckman TJ, Reed DA, Cook DA. **Standards for reporting qualitative research: a synthesis of recommendations.** *Academic Medicine*, Vol. 89, No. 9 / Sept 2014
DOI: 10.1097/ACM.0000000000000388
